# Supplementary material for: KMCP: accurate metagenomic profiling of both prokaryotic and viral populations by pseudo-mapping
Source: Bioinformatics. 2022 Dec 29;39(1):btac845. doi: 10.1093/bioinformatics/btac845 (PMC9828150; doi:10.1093/bioinformatics/btac845)
Supplement: btac845_Supplementary_Data [file btac845_supplementary_data.zip › kmcp-supplementary-material-r2.pdf]

Supplementary Material

KMCP: Accurate metagenomic profiling of prokaryotic and viral populations by pseudo-mapping

Wei Shen[1,*], Hongyan Xiang[1], Tianquan Huang[1], Hui Tang[1], Mingli Peng[1], Dachuan Cai[1], Peng Hu[1,*], Hong Ren[1,*]

[1]Key Laboratory of Molecular Biology for Infectious Diseases (Ministry of Education), Institute for Viral Hepatitis, Department of Infectious Diseases, The Second Affiliated Hospital, Chongqing Medical University, Chongqing 400010, China

*To whom correspondence should be addressed.

Contents

1 Methods 3
1.1 Optimization of searching speed in the modified COBS index 3
1.2 Benchmarking KMCP using the CAMI framework 3
1.3 Benchmarking KMCP using simulated bacterial communities 3
1.4 Benchmarking KMCP using mock virome communities 4
1.5 Benchmarking KMCP using infectious clinical samples 5
1.6 Benchmarking runtime and storage requirements of database building and profiling 5
2 Availability of data and materials 6
Reference 6
3 Tables 8
4 Figures 13

Index of Tables

Table S1. List of KMCP sub-commands 8
Table S2. The three options to create extra smaller blocks 8
Table S3. Comparison of database size and building time on GTDB dataset 8
Table S4. The differences between KMCP and COBS 9
Table S5. Main reference filtering criteria of the six profiling modes 10
Table S6. Summary of KMCP databases 10
Table S7. Summary of species in benchmark datasets 10
Table S8. Accuracies of different profiling modes of KMCP on CAMI2 marine datasets 11
Table S9. Accuracies of different profiling modes of KMCP on simulated bacterial communities 11
Table S10. Accuracies of different profiling modes of KMCP on mock virome communities 11
Table S11. Database size, building time, and memory of KMCP for performance benchmark 12

Index of Figures

Figure S1. Workflow of KMCP subcommands 13
Figure S2. False-positive rates of Bloom filters in index files 14
Figure S3. Benchmarks of searching speed on GTDB dataset 15
Figure S4. *K*-mer similarity vs. percent nucleotide identity 16
Figure S5. False-positive rates of queries 17
Figure S6. Accuracies of KMCP on CAMI mouse gut dataset with different parameters 18
Figure S7. Species abundances in benchmark samples 19
Figure S8. Taxon identification accuracies at the family rank on the mock virome dataset ... 20

1 Methods

1.1 Optimization of searching speed in the modified COBS index

Many optimizations are performed to accelerate batch querying in the modified COBS index. Multiple hashing functions are optionally used to reduce the false-positive rate of Bloom filters, which requires a bitwise AND step to double-check the existence of a *k*-mer. Instead of the byte-by-byte operation performed in COBS, the AND step is optimized with Single-Instruction Multiple-Data (SIMD) instructions. For the ADD step, which cumulatively adds the number of matched *k*-mers in a Bloom filter, COBS uses array lookup to avoid unpacking the bit-packed count information. However, array lookup requires frequent memory access, which is fast but not free. Here, we use a high-performance vectorized positional *popcount* method (https://github.com/clausecker/pospop), which utilizes SIMD instructions to unpack and count *k*-mers in CPU registers for a list of bytes. To balance the speed of memory access for collecting column-wise bytes and invoking the *pospop* function, we set the size of the byte list to 64 (i.e., counting matched elements for 64 *k*-mers in 8 (8 bits for a byte) genome chunks in one function invocation). In addition, sixty-four is the cache line size for most 64-bit computers, which helps to reduce cache misses and provides extra speedup. For the left *k*-mers (<64) of the query sequence, we unroll the loops of 63 possible *k*-mer sizes to increase CPU instruction execution efficiency. Furthermore, many language-level optimizations are performed to reduce memory occupation and accelerate searching speed. A complete list of differences in algorithm and data structure between KMCP and COBS is listed in Table S4.

1.2 Benchmarking KMCP using the CAMI framework

Snapshots of RefSeq and NCBI Taxonomy dump files on Jan 08, 2019 are used to create the KMCP database. TaxIds of archaea and bacteria are retrieved with TaxonKit 0.10.1 (Shen and Ren, 2021) and used to extract genomes from the RefSeq snapshot. The assembly accessions of all genomes are sorted in alphabetical order, and a maximum of five genomes of a species are retained. Since the RefSeq snapshot only contains prokaryotic genomes, we downloaded the viral assembly summary file from RefSeq FTP on Nov 11, 2021, and extracted viral references before Jan 08, 2019. Finally, 9,871 prokaryotic and 8,242 viral genomes are used to build KMCP databases with the parameters described in the "Generation of the KMCP databases" section. The ten marine datasets were downloaded from https://data.cami-challenge.org/participate. KMCP v0.9.0 uses the command "*kmcp search -j 40 -d $db read.fq.gz -o read.kmcp@$db.tsv.gz*" for searching reads against prokaryotic and viral databases, command of "*kmcp merge read.kmcp@*.tsv.gz -o read.kmcp.tsv.gz*" for merging search results, and command of "*kmcp profile -m 3 -X $taxdump -T $taxidmap read.kmcp.tsv.gz -o read.kmcp.tsv.gz.k-m3.profile -C read.kmcp.tsv.gz.c-m3.profile --show-rank superkingdom,phylum,class,order,family,genus,species -s $sampleID*" for profiling. The gold standards and profiles of other tools are downloaded from https://zenodo.org/record/5006866, and OPAL (commit edb6f41 in dev branch) (Meyer *et al.*, 2019) is used to assess taxonomic metagenome profilers. R 4.1.2 (R Core Team, 2022), ggplot2 3.3.5 (Wickham, 2016), and some other R packages are used for data visualization.

1.3 Benchmarking KMCP using simulated bacterial communities

Sun *et al.* (Sun *et al.*, 2021) simulated twenty-five simulated metagenomic sequence reads from five

habitats with genomes selected from the intersection among the reference databases of MetaPhlAn2, mOTUs2, and Kraken2. Each dataset contains 3 Gb of 150-bp paired-end reads. KMCP 0.9.0, with the standard database (Dec 6, 2021); Kraken 2.1.2 and Bracken 2.6.2, with the PlusPF collection of the official databases (May 17, 2021); MetaPhlAn 3.0.13, with the mpa_v30_CHOCOPhlAn_201901 database (July 22, 2020); and mOTUs 3.0.1, with the 2.6.0 database (June 16, 2021), are used for benchmarking. In addition, the genomes for building the KMCP database, including 47,894 prokaryotic genomes from GTDB, 27,936 viral genomes from GenBank, and 403 fungal genomes from RefSeq, are used to create databases for Kraken2/Bracken, Bracken, Centrifuge 1.0.4, Ganon 1.1.2, DUDes 0.08, and SLIMM 0.3.4 with the default parameters. SeqKit 2.1.0 (Shen *et al.*, 2016) is used in reference genome preparation.

KMCP uses the command "*kmcp search -j 40 -d $db read1.fq.gz read2.fq.gz -o read.kmcp@$db.tsv.gz*" for searching reads against prokaryotic and fungal databases, command of "*kmcp merge read.kmcp@*.tsv.gz -o read.kmcp.tsv.gz*" for merging search results, and command of "*kmcp profile -m 3 -X $taxdump -T $taxidmap read.kmcp.tsv.gz -o read.kmcp.tsv.gz.k-m3.profile -C read.kmcp.tsv.gz.c-m3.profile --show-rank superkingdom,phylum,class,order,family,genus,species -s $sampleID*" for profiling. MetaPhlAn3 uses the command "*metaphlan --nproc 40 -x $db --bowtie2db $dbdir --input_type fastq read1.fq.gz,read2.right.fq.gz --bowtie2out bowtie2.bz2 --CAMI_format_output -o mpa3.profile*", and mOTUs3 uses the command "*motus profile -t 40 -f read1.fq.gz -r read2.fq.gz -p -C precision -o motus.profile*". Kraken2 uses the command "*kraken2 --threads 40 --db $db --memory-mapping --gzip-compressed --paired read1.fq.gz read2.fq.gz --report read.kreport > /dev/null*" for classification, and Bracken uses the command "*for r in S G F O C P D; do est_abundance.py -k $db/database150mers.kmer_distrib -l $r -t 10 -i read.kreport -o read.bracken.level-$r; done*" to estimate abundance at species, genus, family, order, class, phylum, and superkingdom ranks, the script tocami.py from CAMI (https://github.com/hzi-bifo/cami2_pipelines/blob/master/bin/tocami.py, commit a0e2ec2) is used to convert the concatenated abundance tables to the CAMI taxonomic profiling format. Centrifuge uses the command "*centrifuge -t -p 40 --mm -x $db -q -1 read1.fq.gz -2 read2.fq.gz --report-file read.cf-report.tsv -S read.cf.tsv*" for classification and uses the command "*centrifuge-kreport -x $db read.cf.tsv > read.kreport*" to generate Kraken-style report, which is further converted to CAMI format with the tocami.py script. Ganon uses the command "*ganon classify -t 40 -d $db -p read1.fq.gz read2.fq.gz -o read*" for classification and the command "*ganon table -l percentage --header taxid -r species -I read.tre -o read.tsv*" to generate the composition table. DUDes and SLIMM use the same command, "*bowtie2 --mm -p 40 -x $bowtie2db --no-unal --very-fast -k 100 -q -1 read1.fq.gz -2 read2.fq.gz -S read.sam*", for reads mapping. DUDes uses the command "*DUDes.py -t 1 -s read.sam -d $db -o read*", and SLIMM uses the command "*slimm -w 1000 -o read $db read.sam*" for profiling. The taxonomic profiles of the gold standards and all taxonomic profiles were converted to CAMI format with the NCBI taxonomy dump file of Dec 6, 2021, and the virus predictions were filtered using TaxonKit 0.10.1.

1.4 Benchmarking KMCP using mock virome communities

Roux *et al*. (Roux *et al.*, 2016) designed two mock viral communities, including two ssDNA viruses and ten dsDNA viruses. Each virus was grown on its specific host (*Pseudoalteromonas*, *Cellulophaga baltica,* or *Escherichia coli*), and viral capsids were obtained from lysates and mixed. Sixteen datasets were generated with three sequencing libraries. The clean data contained paired

and unpaired reads with lengths of 50 to 250 bp, and the amount of data from each sample ranged from 900 Mb to 2.5 Gb. The normalized coverage of each genome was used as a proxy for the relative abundance of each viral genome (Roux *et al.*, 2016). mOTUs3, without virus detection support, was not included in this benchmark. MetaPhlAn3 was run with the flag "--*add_viruses*" to add virus detection. KMCP v0.9.0 searches against prokaryotic, fungal, and viral databases. After the initial community composition analysis, nonviral predictions were filtered out using TaxonKit 0.10.1.

Reads are assembled with SPAdes 3.15.5 (Nurk *et al.*, 2017) with the metagenomic modes (--meta) and a *k*-mer size of 55, and contigs shorter than 2 kb are filtered out. VIRify 0.4.0 (Rangel-Pineros *et al.*, 2022) is used to perform virus detection and annotation from the contigs with the command of "*nextflow run EBI-Metagenomics/emg-viral-pipeline -r v0.4.0 --databases $db --cachedir $cache --ncbi $ete3tax --fasta $contigs --workdir work --output virify --length 2 -profile local,singularity*" with the same taxonomy data (NCBI taxonomy dump file of Dec 6, 2021,) to KMCP. DeepVirFinder v1.0 (Ren *et al.*, 2020) is used to identify viral sequences with the default parameters and then PhaGCN (commit 5c40b59) (Shang *et al.*, 2021) is used to annotate viral sequences with the default parameters. At last, the accuracies of taxon identification at the family rank are computed.

1.5 Benchmarking KMCP using infectious clinical samples

Gu *et al.* (Gu *et al.*, 2021) collected 182 body fluid samples from 160 patients as residual samples after routine clinical testing in the microbiology laboratory, among which 87 have accessible short-read datasets and have been verified by culture or 16S rRNA gene qPCR. The number of reads in the clean data ranges from 49 to 252,311 (Table S3). Kraken2 and Bracken, Centrifuge, and KMCP are used to detect pathogens, and all tools require a minimum of two reads. KMCP uses a preset profiling mode with the highest sensitivity for pathogen detection, in which the predictions are sorted in reverse order by the product of the genome chunk fraction and the similarity score, rather than the read number or taxonomic abundance, as in other tools. The score is the 90th percentile of *k*-mer coverage of all uniquely matched reads. For Bracken, the number of new estimated reads (column *new_est_reads*) is used for sorting. The number of reads (column *numReads*) is used in Centrifuge. Similarly, the taxon abundances in the Ganon results are sorted by the number of reads.

1.6 Benchmarking runtime and storage requirements of database building and profiling

The performance of KMCP and other tools is analyzed on a server with four Intel(R) Xeon(R) Gold 6248 processors (80-CPUs/160-threads) and one terabyte of main memory, and the reference genomes and databases are stored on solid-state disks, while the sequencing reads are saved on hard disk drives. The operation system is Centos 7.9.2009 with a kernel of 3.10.0-1160.11.1.

The genomes used for building the KMCP databases, including 47,894 prokaryotic genomes from GTDB, 27,936 viral genomes from GenBank, and 403 fungal genomes from RefSeq, are used to create databases for Kraken, Bracken, and Centrifuge with the default parameters. MetaPhlAn3 and mOTUs3 are tightly associated with the official databases, and the most recent databases (mpa_v30_CHOCOPhlAn_201901 for MetaPhlAn3 and 3.0.1 for mOTUs3) are used. Eight samples from the CAMI mouse gut metagenome datasets are used to assess the analysis time and memory requirement. All tools use 40 threads for database building and taxonomic profiling. The

scaling test of KMCP is performed on a computer cluster with 24 nodes, each with one Hygon C86 7185 32-core processor and 124 GB of memory. A script (https://github.com/shenwei356/easy_sbatch) is used for the batch submission of search jobs against all databases.

2 Availability of data and materials

The CAMI2 marine datasets, mouse gut datasets, and the Jan 8, 2019 RefSeq snapshot can be downloaded from https://data.cami-challenge.org/participate, and the gold standard taxonomic profile and profiles of other tools can be downloaded from https://zenodo.org/search?page=1&size=20&q=keywords:%22CAMI%202%20Mouse%20Gut%20Toy%20data%20set%22&keywords=taxonomic%20profiling. The reference genomes and database used by KMCP can be downloaded from https://1drv.ms/u/s!Ag89cZ8NYcqtjVVADr8r--fnKFt-?e=ivNZNK. Simulated metagenomic sequence reads from Sun *et al*. (Sun *et al.*, 2020) can be downloaded from https://figshare.com/projects/Pitfalls_and_Opportunities_in_Benchmarking_Metagenomic_Classifiers/79916, and the gold standard profiles in CAMI format can be downloaded from https://github.com/shenwei356/sun2021-cami-profiles/releases/tag/v2021-12-06. Mock virome communities A and B from Roux *et al*. (Roux *et al.*, 2016) can be downloaded from https://datacommons.cyverse.org/browse/iplant/home/shared/iVirus/DNA_Viromes_library_comparison, and the gold standard profiles in CAMI format can be downloaded from https://github.com/shenwei356/roux2016-mock-virome-cami-profile/releases/tag/v2021-12-06. The metagenomic samples of infected body fluids from Gu *et al*. (Gu *et al.*, 2021) can be downloaded from https://www.ncbi.nlm.nih.gov/bioproject/?term=PRJNA558701, and the list of run accessions can be downloaded from https://github.com/shenwei356/kmcp/blob/cb98c64c7dda4f1863c603a6dd38296572479396/benchmarks/real-pathogen-gu2020/acc2sample.tsv.

The source code of KMCP is under the MIT license and is publicly available on Github (https://github.com/shenwei356/kmcp) and Zenodo (https://zenodo.org/record/7117838). The prebuilt databases can be downloaded from https://1drv.ms/u/s!Ag89cZ8NYcqtjHwpe0ND3SUEhyrp?e=QDRbEC. Benchmarking steps, generated taxonomic profiles, plotting scripts, and other data generated and analyzed during the current study are available in the Zenodo repository (https://doi.org/10.5281/zenodo.7450803).

Reference

Gu, W., *et al.* (2021) Rapid pathogen detection by metagenomic next-generation sequencing of infected body fluids. Nat Med, **27**(1), 115-124.

Meyer, F., *et al.* (2019) Assessing taxonomic metagenome profilers with OPAL. Genome Biol, **20**(1), 51.

Nurk, S., *et al.* (2017) metaSPAdes: a new versatile metagenomic assembler. Genome Res, **27**(5), 824-834.

R Core Team. 2022. R: A Language and Environment for Statistical Computing. https://www.R-

project.org/
Rangel-Pineros, G., *et al.* (2022) VIRify: an integrated detection, annotation and taxonomic classification pipeline using virus-specific protein profile hidden Markov models. bioRxiv, 2022.2008.2022.504484.
Ren, J., *et al.* (2020) Identifying viruses from metagenomic data using deep learning. Quant Biol, **8**(1), 64-77.
Roux, S., *et al.* (2016) Towards quantitative viromics for both double-stranded and single-stranded DNA viruses. PeerJ, **4**, e2777.
Shang, J., *et al.* (2021) Bacteriophage classification for assembled contigs using graph convolutional network. Bioinformatics, **37**(Suppl_1), i25-i33.
Shen, W., *et al.* (2016) SeqKit: A Cross-Platform and Ultrafast Toolkit for FASTA/Q File Manipulation. PLoS One, **11**(10), e0163962.
Shen, W. and Ren, H. (2021) TaxonKit: A practical and efficient NCBI taxonomy toolkit. J Genet Genomics, **48**(9), 844-850.
Sun, Z., *et al.* (2020) Challenges in Benchmarking Metagenomic Profilers. bioRxiv, 2020.2011.2014.382994.
Sun, Z., *et al.* (2021) Challenges in benchmarking metagenomic profilers. Nat Methods, **18**(6), 618-626.
Wickham, H. ggplot2: Elegant Graphics for Data Analysis. Springer-Verlag New York; 2016.

3 Tables

Table S1. List of KMCP sub-commands.

Sub-command	Description
`compute`	Generating *k*-mers (sketches) from FASTA/Q sequences
`index`	Constructing database from *k*-mer files
`search`	Searching sequences against a database
`merge`	Merging search results from multiple databases
`profile`	Generating taxonomic profile from search results
`utils split-genomes`	Splitting genomes into chunks
`utils unik-info`	Printing information of .unik file
`utils index-info`	Printing information of index file
`utils ref-info`	Printing information of reference chunks in a database
`utils cov2simi`	Convert *k*-mer coverage to sequence similarity
`utils query-fpr`	Compute the maximal false positive rate of a query
`utils filter`	Filtering search results and finding species/assembly-specific queries
`utils merge-regions`	Merging species/assembly-specific regions

Table S2. The three options to create extra smaller blocks. Uneven genome size distribution would make Bloom filters of the last block with the most *k*-mers extremely huge, so three thresholds are used to split the last block.

Option	Default threshold	Size of block	Description
-x/--block-sizeX-kmers-t	10 M	256 (settable)	If the number of *k*-mers of an .unik file exceeds the threshold, the block size is changed to 256.
-8/--block-size8-kmers-t	20 M	8	If the number of *k*-mers of an .unik file exceeds the threshold, the block size is changed to 8.
-1/--block-size1-kmers-t	200 M	1	If the number of *k*-mers of an .unik file exceeds the threshold, an individual index is created for this file.

Table S3. Comparison of database size and building time on GTDB dataset. The 47,894 bacteria and archaea genomes from GTDB r202 representative collection were used to build databases. COBS and KMCP used the same parameters of k = 31, block size = 1024, hash functions = 1, and false-positive rate of Bloom filters = 0.3.

Tool	Database size	Building time	Temporary files size
COBS	86.96 GB	29m 55s	**160.76 GB**
KMCP	**55.15 GB**	**21m 04s**	935.11 GB

Table S4. The differences between KMCP and COBS.

Category	Item	COBS	KMCP	Comment
Algorithm	*K*-mer hashing	xxhash	ntHash	The xxHash is a general-purpose hashing function, while the ntHash is a recursive hash function for DNA/RNA
	Bloom filter hashing	xxhash	Using *k*-mer hash values	Avoid hash computation
	Multiple-hash functions	xxhash with different seeds	Generating multiple values from a single one	Avoid hash computation
	Single-hash function	Same as multiple-hash functions	Separated workflow	Reducing loops
	AND step	Serial bitwise AND	Vectorized bitwise AND	Bitwise AND for >1 hash functions
	PLUS step	Serial bit-unpacking	Vectorized positional popcount with pospop	Counting from bit-packed data
Index structure	Size of blocks	/	Using extra thresholds to split the last block with the most *k*-mers	Uneven genome size distribution would make Bloom filters of the last block extremely huge
	Index files	Concatenated	Independent	Index files
	Index loading	mmap, loading complete index into RAM	mmap, loading complete index into RAM, seek	Index loading
Input/ output	Input files	FASTA/Q, McCortex, text	FASTA/Q	Input files
	Output	Target and matched *k*-mers	Target, matched *k*-mers, query FPR, etc.	Output

Table S5. Main reference filtering criteria of the six profiling modes.

Filtering criteria	m0	m1	m2	m3 (default)	m4	m5
Minimum reads of a chunk	1	5	10	50	100	100
Minimum chunk fraction	0.2	0.6	0.7	0.8	1	1
Maximum standard deviation of the relative depth of genome chunks	10	2	2	2	2	1.5
Minimum uniquely matched reads	1	2	5	20	50	50
Minimum high-confidence uniquely matched reads	1	1	2	5	10	10
Minimum *k*-mer coverage of high-confidence uniquely matched reads	0.7	0.7	0.7	0.75	0.8	0.8
Minimum proportion of high-confidence uniquely matched reads	0.01	0.1	0.2	0.1	0.1	0.15
Keeping main matches	Yes					
Maximum query coverage gap	0.4					

Table S6. Summary of KMCP databases.

Database	Source	#species	#assemblies	*k*	Chunks	FPR	Size (GB)
Bacteria and Archaea	GTDB r202	28073	47894	21	10	0.3	58.03
Fungi	Refseq r208	398	403	21	10	0.3	4.18
Viruses	GenBank r246	23632	27936	21	10	0.3	4.72

Table S7. Summary of species in benchmark datasets, including the number of species and the percentage of abundance.

Dataset	Environment	Minimum #species	Maximum #species	Minimum %abundance	Maximum %abundance
CAMI marine datasets	/	256	381	0.0010	11.5330
Simulated prokaryotic communities	Building	90	113	0.0370	15.7443
	Gut	56	76	0.0277	19.2673
	Oral	73	98	0.0364	12.2192
	Skin	52	80	0.0504	14.9236
	VG	21	38	0.1067	48.0633
Mock virome datasets	/	10	12	0.0020	74.9248

Table S8. Accuracies of different profiling modes of KMCP on CAMI2 marine datasets.

Rank	Filtering criteria	m1	m2	m3	m4	m5
Genus	Completeness	**0.9449**	0.9388	0.9358	0.9274	0.9219
	Purity	0.9367	0.9528	0.9551	0.9562	**0.9559**
	F1 score	0.9408	**0.9458**	0.9454	0.9415	0.9385
	L1 norm error	**0.1585**	0.1626	0.1611	0.1828	0.2153
Species	Completeness	**0.9298**	0.9200	0.9151	0.9037	0.8955
	Purity	0.7488	0.8202	0.8303	0.8739	**0.8976**
	F1 score	0.8294	0.8672	0.8705	0.8885	**0.8964**
	L1 norm error	0.2639	0.2650	0.2653	0.2649	**0.2665**
/	Weighted UniFrac error	0.3191	0.3217	**0.3183**	0.3346	0.3573

Table S9. Accuracies of different profiling modes of KMCP on simulated bacterial communities.

Rank	Filtering criteria	m1	m2	m3	m4	m5
Genus	Completeness	**1.0000**	0.9967	0.9926	0.9748	0.9687
	Purity	0.8238	0.9168	0.9667	0.9857	0**.9875**
	F1 score	0.9021	0.9547	0.9794	**0.9802**	0.9779
	L1 norm error	**0.1287**	0.1356	0.1345	0.1604	0.1927
Species	Completeness	**0.9904**	0.9765	0.9806	0.9440	0.9062
	Purity	0.4851	0.6656	0.8033	0.9018	**0.9282**
	F1 score	0.6500	0.7902	0.8823	**0.9221**	0.9169
	L1 norm error	**0.2417**	0.2492	0.2453	0.2728	0.3090
/	Weighted UniFrac error	**0.1617**	0.1710	0.1696	0.2035	0.2509

Table S10. Accuracies of different profiling modes of KMCP on mock virome communities.

Rank	Filtering criteria	m1	m2	m3	m4	m5
Genus	Completeness	**0.9143**	**0.9143**	0.9000	0.8821	0.8821
	Purity	0.9536	0.9714	**1.0000**	**1.0000**	**1.0000**
	F1 score	0.9263	0.9365	**0.9444**	0.9342	0.9342
	L1 norm error	0.0889	0.0889	**0.0875**	0.1532	0.1532
Species	Completeness	**0.9311**	**0.9311**	0.9073	0.8769	0.8769
	Purity	0.8441	0.8669	0.8757	**0.9044**	**0.9044**
	F1 score	0.8839	**0.8966**	0.8906	0.8898	0.8898
	L1 norm error	0.1501	**0.1480**	0.1762	0.2732	0.2732
/	Weighted UniFrac error	0.0848	**0.0846**	0.0885	0.1148	0.1148

Table S11. Database size, building time, and memory of KMCP for performance benchmark.

Database	Command	Index size	Building time	Peak memory
Bacteria and Archaea	compute	/	9m 01s	4.81 GB
	index	56.94 GB	10m 47s	11.02 GB
Fungi	compute	/	59.405s	14.44 GB
	index	4.16 GB	44.349s	1.36 GB
Viruses	compute	/	22.920s	4.54 GB
	index	4.33 GB	28.894s	2.87 GB

4 Figures

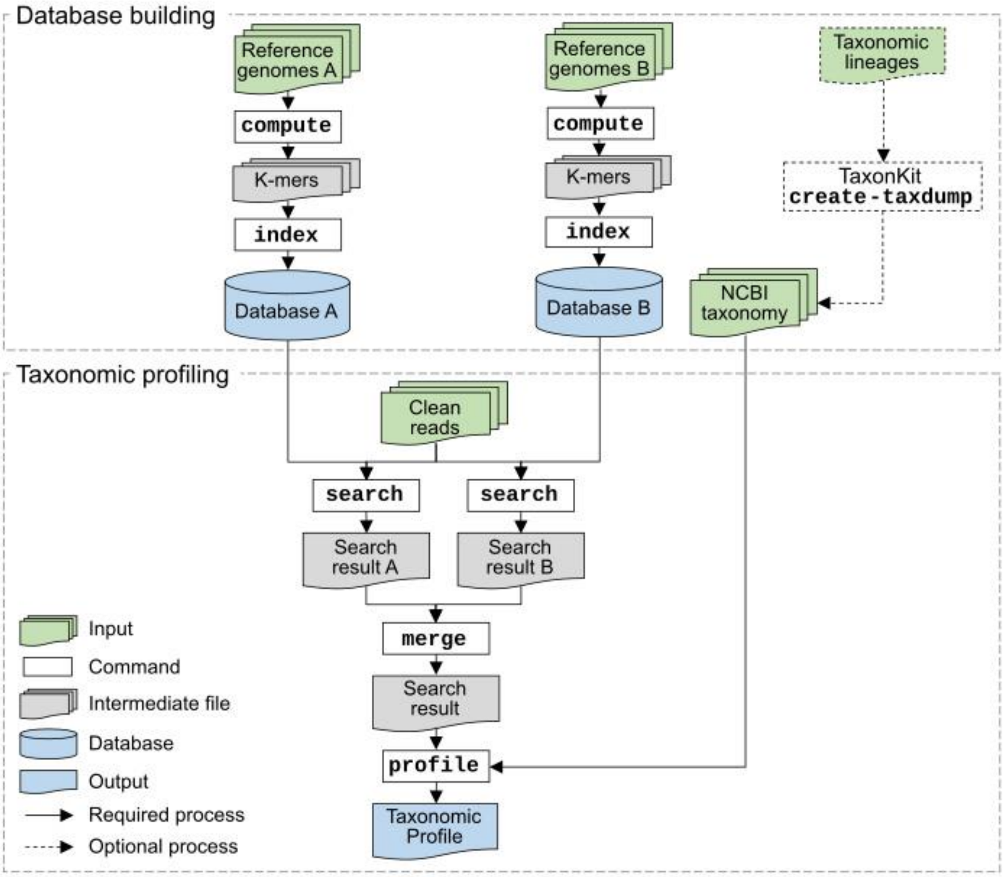

Figure S1. Workflow of KMCP subcommands.

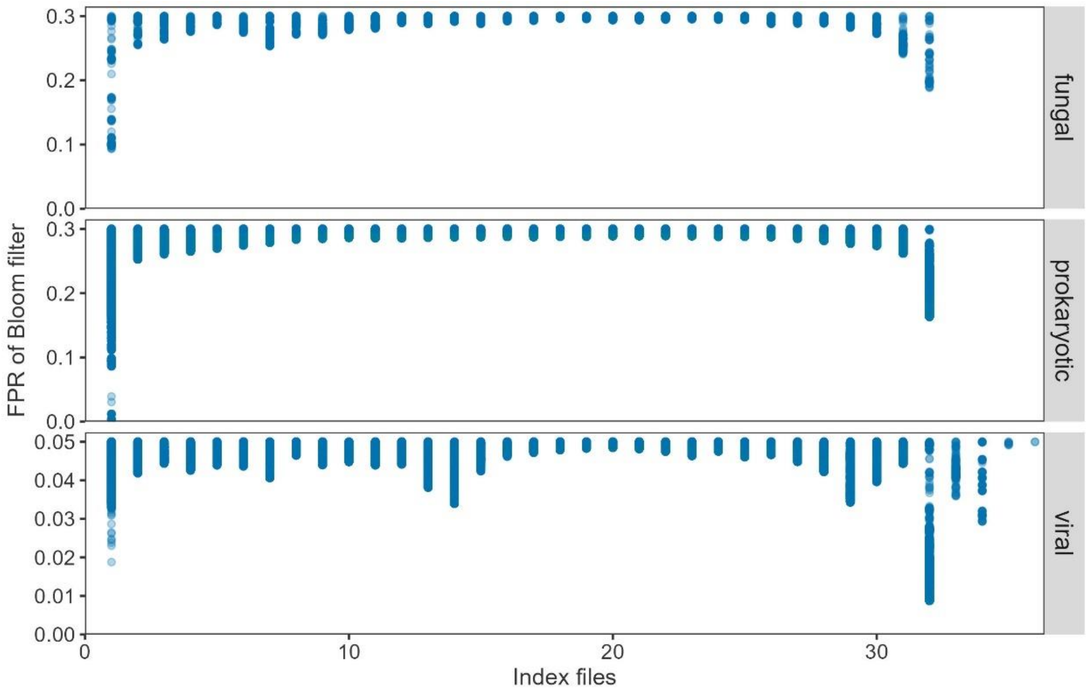

Figure S2. **False-positive rates of Bloom filters in index files**. The defined false-positive rate of an index block is valid only for the Bloom filter with the most *k*-mers, and other Bloom filters have the same or lower false-positive rate due to fewer *k*-mers. The defined false-positive rate of the viral database is set to 0.05 in consideration of the smaller genome size.

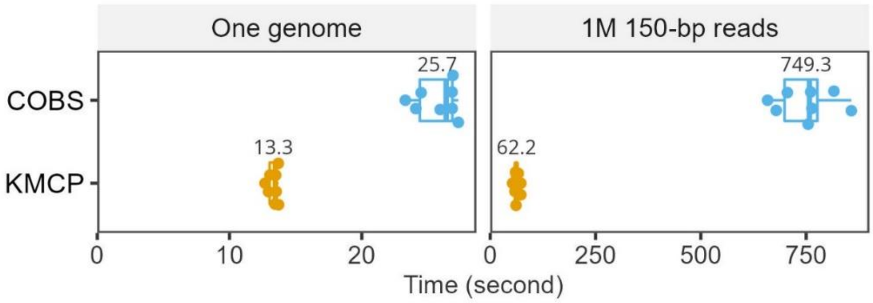

Figure S3. Benchmarks of searching speed on GTDB dataset. The 47,894 bacterial and archaeal genomes from GTDB r202 representative collection are used to build databases with the whole *k*-mers (k = 31), and eight bacterial genomes (4-6 Mb) are used as queries. The complete genomes (left) or short reads (right) generated from the genomes are used for searching with 40 threads using COBS (commit 1915fc0) and KMCP v0.9.0. The searches are performed twice, and metrics of the second run (hot start, with index data cached in RAM) are used for plotting, with each dot representing one query data. The accessions of query bacterial genomes are NC_000913.3, NC_002695.2, NC_011750.1, NC_012971.2, NC_013654.1, NC_018658.1, NZ_CP007592.1, and NZ_CP028116.1. Reads are generated by sliding genomes with a window of 150 bp and a step of 4 bp using SeqKit.

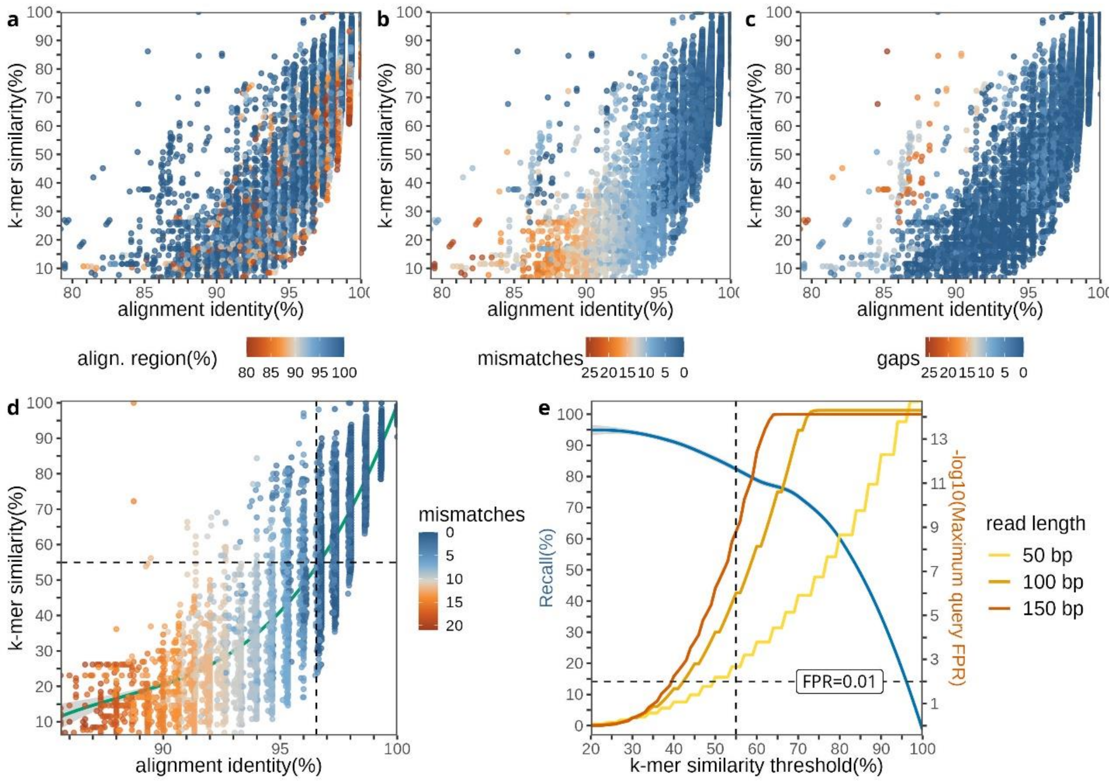

Figure S4. *K*-mer similarity vs. percent nucleotide identity. Simulated 150-bp reads are generated from five *Escherichia coli* genomes (GCA_000613265.1, GCA_900706755.1, GCF_000690815.1, GCF_000734955.1, GCF_003697165.2), and aligned to all the genomes with BLASTN. Conserved across all strains but imperfect matches with the percentage of alignment region ≥ 80% are kept, with the best one hit for a search subject. The *k*-mer similarity (percentage of matched *k*-mers for a read) and the alignment identity along with the percentage of the aligned region **(a)**, the number of mismatches **(b)**, and the length of gaps **(c)** are plotted. **(d)** Matches are further filtered with percent identity > 85%, gap length ≤ 5, and the percentage of aligned regions ≥ 95%. Then a polynomial model of degree 3 is fitted. **(e)** Correlation between *k*-mer similarity threshold and recall of matches and the false-positive rate for reads searching against a KMCP database constructed with one hash function and false-positive rate of 0.3 for each Bloom filter.

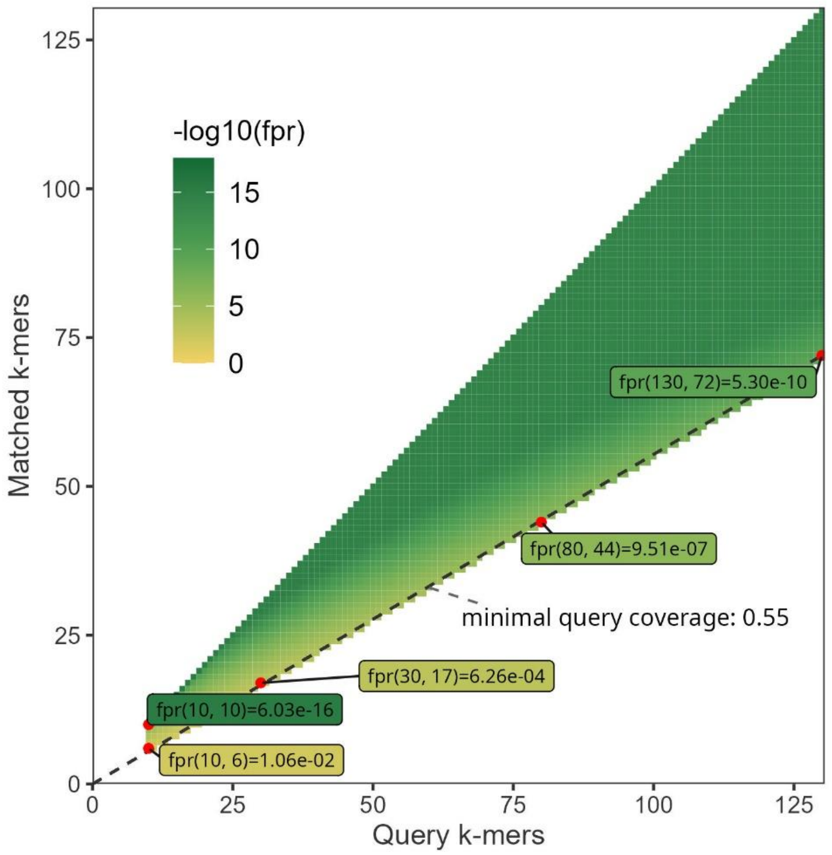

Figure S5. False-positive rates of queries. A minimum *k*-mer ($k = 21$) coverage of 0.55 is required for reads ranging from 30 bp to 150 bp. The false-positive rate (FPR) of a single *k*-mer is 0.3, and FPRs of all possible matches are computed according to Theorem 2 in the [Solomon and Kingsford paper,] which is also implemented in *query-fpr* command in KMCP. For example, a read of 100 bp with 80 (100-21+1) *k*-mers has 44 *k*-mer matched; then the FPR is 9.51×10^{-7}.

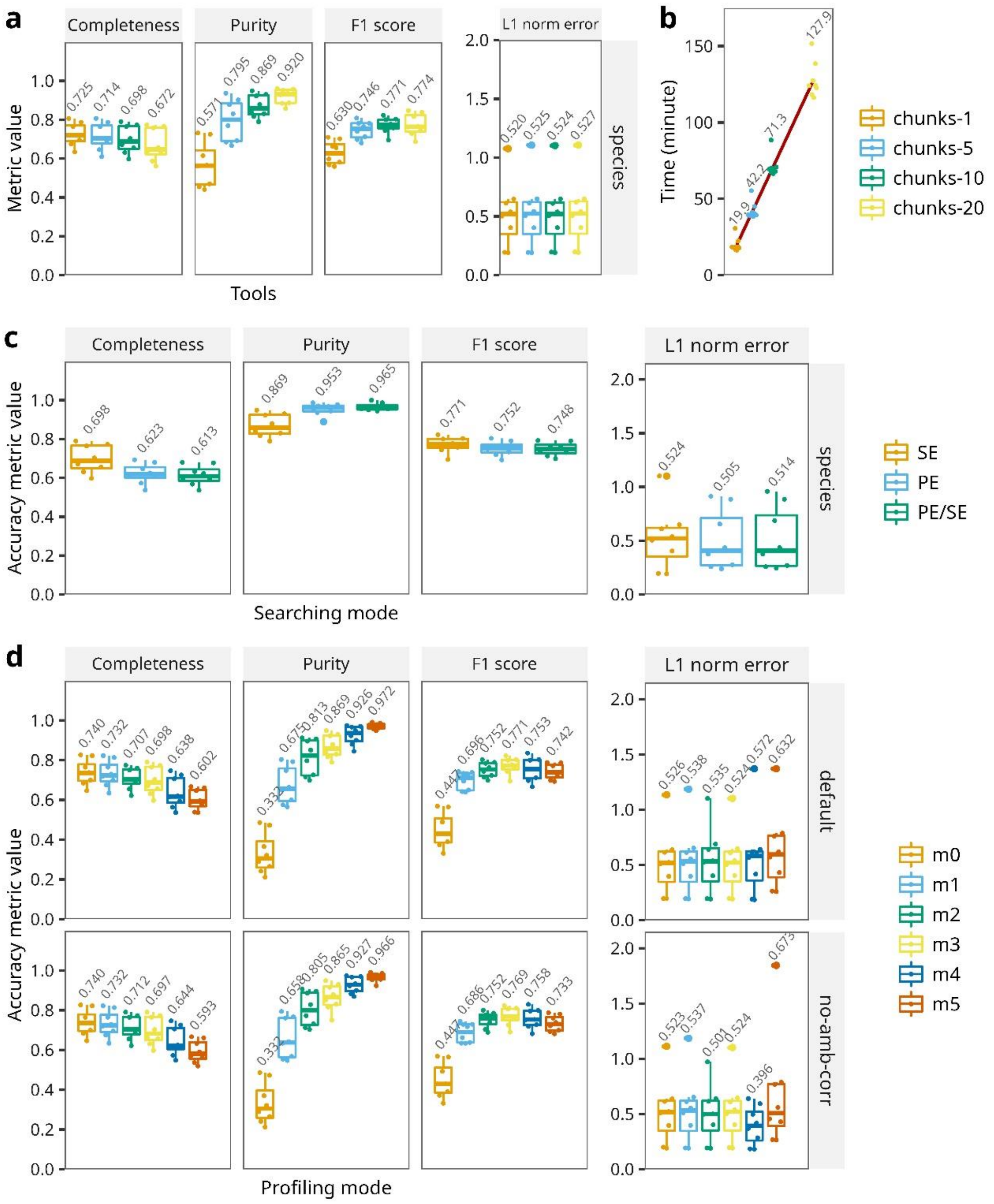

Figure S6. Accuracies of KMCP on CAMI mouse gut dataset with different parameters. (a) Snapshots of RefSeq and NCBI Taxonomy dump files on Jan 08, 2019 are used to create the KMCP database with genome chunks of 1, 5, 10, and 20, respectively. And eight simulated mouse gut datasets are used to perform taxonomic profiling with the four databases, respectively. The running time, including the searching and profiling steps, is shown in **(b). (c)** Reference genomes are split into ten chunks, and query reads are searched as single-end (SE), paired-end (PE), or paired-end OR single-end (PE/SE) where PE reads are searched as SE if there are no matches when searching as PE. **(d)** Reference genomes are split into ten chunks, and taxonomic profiling is performed with the six profiling modes respectively. "no-amb-corr" means the flag "—no-amb-corr" is switched on where the two-stage taxonomy assignment algorithm in MegaPath is not used.

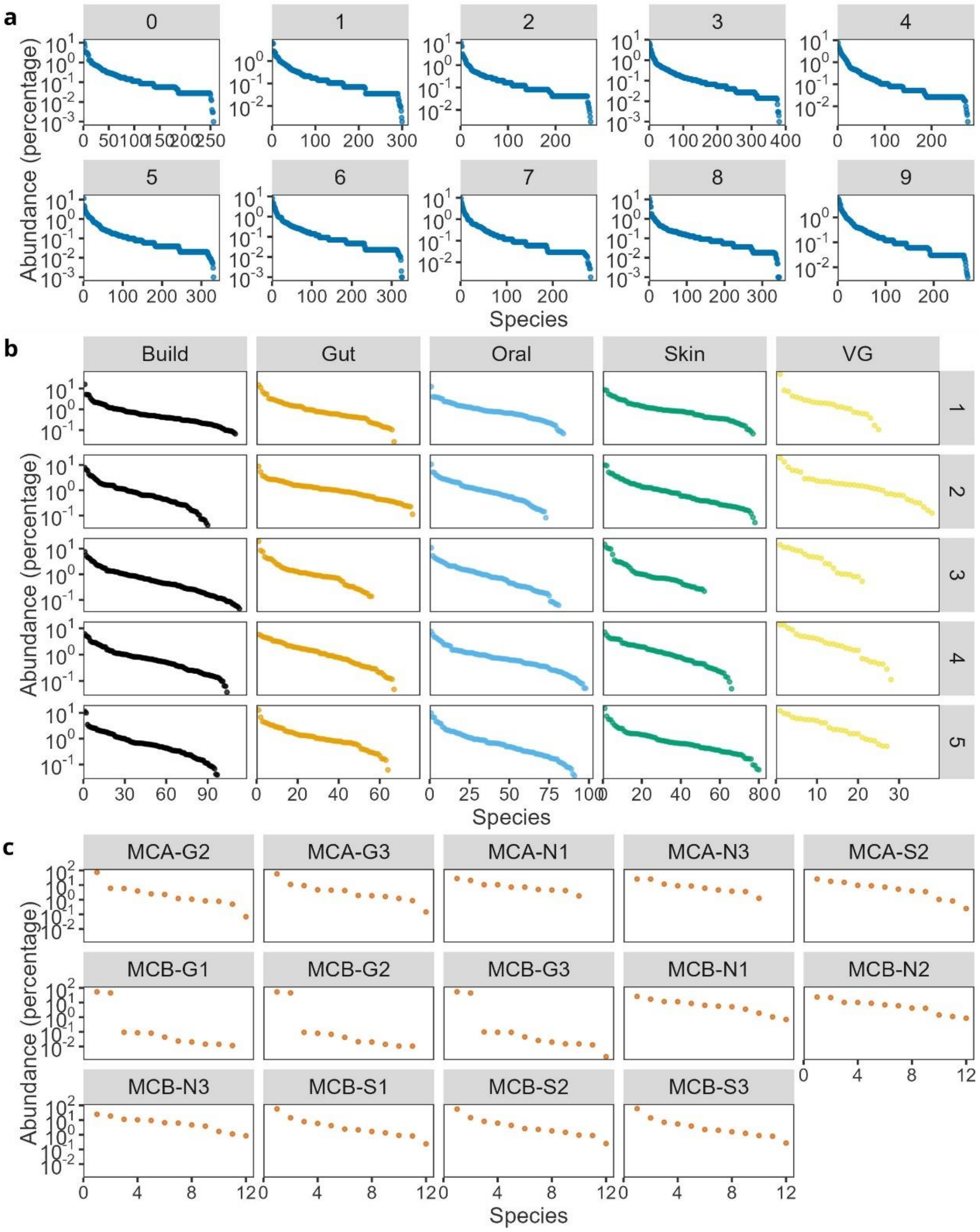

Figure S7. **Species abundances in benchmark samples**. Species of the reference genomes are sorted in descending order of percentage of taxonomic abundance. **(a)** The CAMI marine datasets. **(b)** The simulated prokaryotic communities. **(c)** The mock virome datasets.

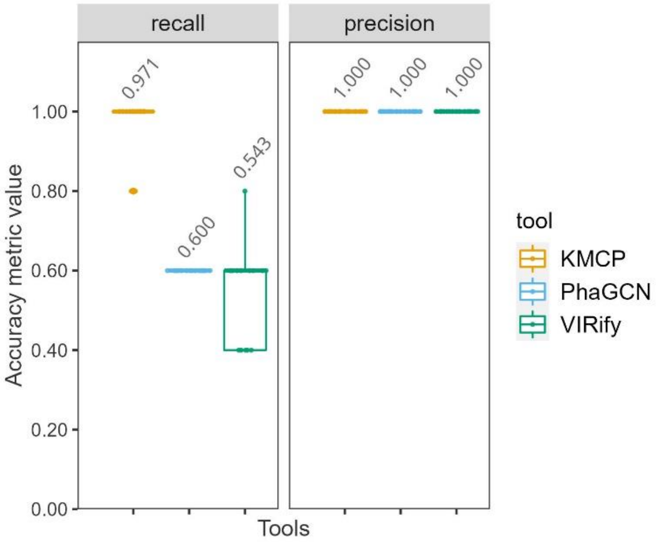

Figure S8. Taxon identification accuracies at the family rank on the mock virome dataset. The lowest rank supported is Family for PhaGCN and Genus for VIRify. Therefore, we compared taxon identification accuracies at the family rank.
